# Distinct mechanisms of acquisition of *mcr-1* –bearing plasmid by *Salmonella* strains recovered from animals and food samples

**DOI:** 10.1038/s41598-017-01810-4

**Published:** 2017-10-16

**Authors:** Mingquan Cui, Jinfei Zhang, Chunping Zhang, Ruichao Li, Edward Wai-chi Chan, Chenbin Wu, Congming Wu, Sheng Chen

**Affiliations:** 1 Beijing Advanced Innovation Center for Food Nutrition and Human Health, College of Veterinary Medicine, China Agriculture University, Beijing, China; 2 Shenzhen Key lab for Food Biological Safety Control, Food Safety and Technology Research Center, Hong Kong PolyU Shenzhen Research Institute, Shenzhen, P. R. China; 3 State Key Lab of Chirosciences, Department of Applied Biology and Chemical Technology, The Hong Kong Polytechnic University, Hung Hom, Kowloon Hong Kong; 4 China Institute of Veterinary Drug Control, Beijing, P. R. China

## Abstract

Since the report of its discovery in *E. coli* in late 2015, the plasmid-mediated colistin resistance gene, *mcr-1*, has been detected in various bacterial species in clinical setting and various environmental niches. However, the transmission mechanisms of this gene in *Salmonella* is less defined. In this study, we conducted a comprehensive study to characterize the genetic features of *mcr-1*-positive *Salmonella* strains isolated from animals and foods. Our data revealed that *Salmonella* recovered from animals and food specimens exhibited highly different PFGE patterns, and acquired *mcr-1*-encoding plasmids via different mechanism. Plasmids harboring *mcr-1* in *Salmonella* food isolates were all conjugative and similar as plasmids reported in other species of Enterobacteriaceae, whereas *mcr-1*-bearing plasmids from animal *Salmonella* isolates were not conjugative, and belonged to the IncHI2 type. The lack of a region carrying the *tra* genes was found to account for the inability to undergo conjugation for various sizes of IncHI2 plasmids harbored by animal strains. These data suggest that transmission of *mcr-1*-positive *Salmonella* from animal to food might not be a common event and food isolates may have acquired *mcr-1*-bearing plasmids from other *mcr-1*-positive bacteria such as *E. coli*, which co-exist in food samples.

## Introduction

Since the discovery of the mobile colistin resistance determinant *mcr-1* in *E. coli*, it has been reported in almost all parts of the world and in many different species of bacteria^1–7^. It was speculated that widespread dissemination of this resistance element may be due to the extensive use of polymyxin as growth promoter in animals, imposing an enormous selection pressure on this genetic element in Enterobacteriaceae in particular *E. coli*. It is important to know the prevalence and transmission of *mcr-1* in key Enterobacteriaceae species such as *Salmonella*, which is an important human pathogen prevalent in animal GI tract in particular chicken and pigs. The *mcr-1* gene was first reported in *Salmonella* through analysis of WGS sequence available in GenBank, in which *mcr-1*-bearing plasmids were identified in 10 clinical *S*. enterica isolates submitted between 2012 and 2015, including 8*S*. Typhimurium, 1*S*. Paratyphi B var Java and 1*S*. Virchow strains^8^. The *mcr-1* gene was subsequently reported to be recoverable from *Salmonella* strains isolated from food, animals and clinical specimens in Europe, the US and China^9–15^. Three types of *mcr-1-*carrying plasmids including IncX4, IncI1and IncHI2 types of plasmids of various sizes have been reported in *Salmonella* ^9,11,12^. However, these data remain scattered, and do not provide a comprehensive view on the prevalence of the *mcr-1* gene in *Salmonella* or the transmission kinetics of the different types of mobile elements that harbor such resistance determinant. To address these important issues, we investigated the prevalence of the *mcr-1* gene in *Salmonella* strains recovered from animals and food collected nationwide in China and investigated the transmission potential of the *mcr-1* gene recovered from two different categories of *Salmonella* isolates. Our data provided a comprehensive view of the extent of *mcr-1* contamination in *Salmonella* in animals and food in China, and essential insights into the transmission mechanism of *mcr-1* in *Salmonella*.

## Results

### Prevalence of *mcr-1* in *Salmonella* isolated from animals and food samples

A total of 1312 non-repeated *Salmonella* isolates were recovered from animal fecal samples in various regions of China during a four-years Nationwide *Salmonella* Antimicrobial Resistance Surveillance Program (2012–2015) conducted in the China Institute of Veterinary Drug Control. A total of 175 *Salmonella* isolated were obtained from 1914 animal fecal samples in 2012; 290 isolates were obtained from 3205 animal fecal samples in 2013; 333 isolates were obtained from 2980 fecal samples in 2014 and 514 were obtained from 3586 fecal samples in 2015. The animal fecal samples were collected from Shanghai, Sichuan, Shandong, Guangdong, Inner Mongolia, Liaoning, Henan, Hunan, Hubei, Yunnan, Chongqing, Shaanxi, Shanxi, Jiangxi, Jilin, Hebei, Guangxi, Fujian, Beijing and Anhui provinces or municipalities. Among these 1312 *Salmonella* isolates, 569 isolates were from chicken fecal samples and 743 were from pig fecal samples. The *mcr-1* gene screening for these 1312 *Salmonella* isolates showed that 26 (2%) *Salmonella* isolates were positive to *mcr-1*, among which 19 were from 2013 and 7 were from 2015. No *mcr-1*-positive *Salmonella* isolate was detected in *Salmonella* isolates collected in 2012 and 2014. A total of 830 *Salmonella* strains isolated from various of food samples including chicken, pork, beef and seafood in Shenzhen in 2015, and 230 food-borne *Salmonella* isolates collected from various provinces and submitted to the China CDC during the period 2012~2015 were tested. Twelve out of 830 (1.4%) *Salmonella* isolates collected from Shenzhen were positive for *mcr-1*; the corresponding positive rate for the CDC samples was 1.3% (4 out of 230).

### Antimicrobial susceptibility and serotype distribution of *mcr-1*-positive *Salmonella*

All *mcr-1* positive *Salmonella* isolates were subjected to determination of their susceptibilities to 14 antibiotics, the results of which were shown in Table 1. These isolates generally displayed a high rate of resistance to other antibiotics, in addition to colistin, yet substantial variations between strains recovered from animal and food were observed. Animal isolates exhibited a very high rate of resistance to most of test agents, while none of the *Salmonella* strains in this category were resistant to ceftriaxone. In contrast, 13% of *Salmonella* isolates from food were resistant to ceftriaxone, while the resistance rate to other antibiotics tested was relatively low compared to organisms recovered from animals (Table 1).

**Table 1:** Antimicrobial susceptibility of *mcr-1*-positive *Salmonella* strains isolated from different sources.

Antibiotics	Break-point (µg/ml)	% resistance		
		Food isolates		
		WT (n = 15)	TC (n = 15)	Animal isolates (n = 26)
Ampicillin	≥32	94	94	100
Amoxicillin/Clavulanic acid	≥32/16	13	13	62
Ceftriaxone	≥4	13	13	0
Ceftazidime	≥4	13	13	0
Cefotaxime	≥4	13	13	0
Meropenem	≥4	13	13	0
Chloramphenicol	≥32	75	13	96
Gentamicin	≥16	31	26	100
Nalidixic acid	32	38	0	96
Ciprofloxacin	≥1	25	0	100
Trimethoprim/ Sulfamethoxazole	≥4/76	44	17	96
Tetracycline	≥16	100	0	100
*Colistin	≥4	100	100	100
Azithromycin	≥16	0	0	0

WT, wild type; TC, transconjugants;

*there is no CLSI breakpoint for colistin in Enterobacteriaceae. The forthcoming 2017 CLSI guideline proposed it to be 4 µg/ml.

### Serotypes and genetic relatedness of *mcr-1*-positive *Salmonella* strains

**Figure 1:**
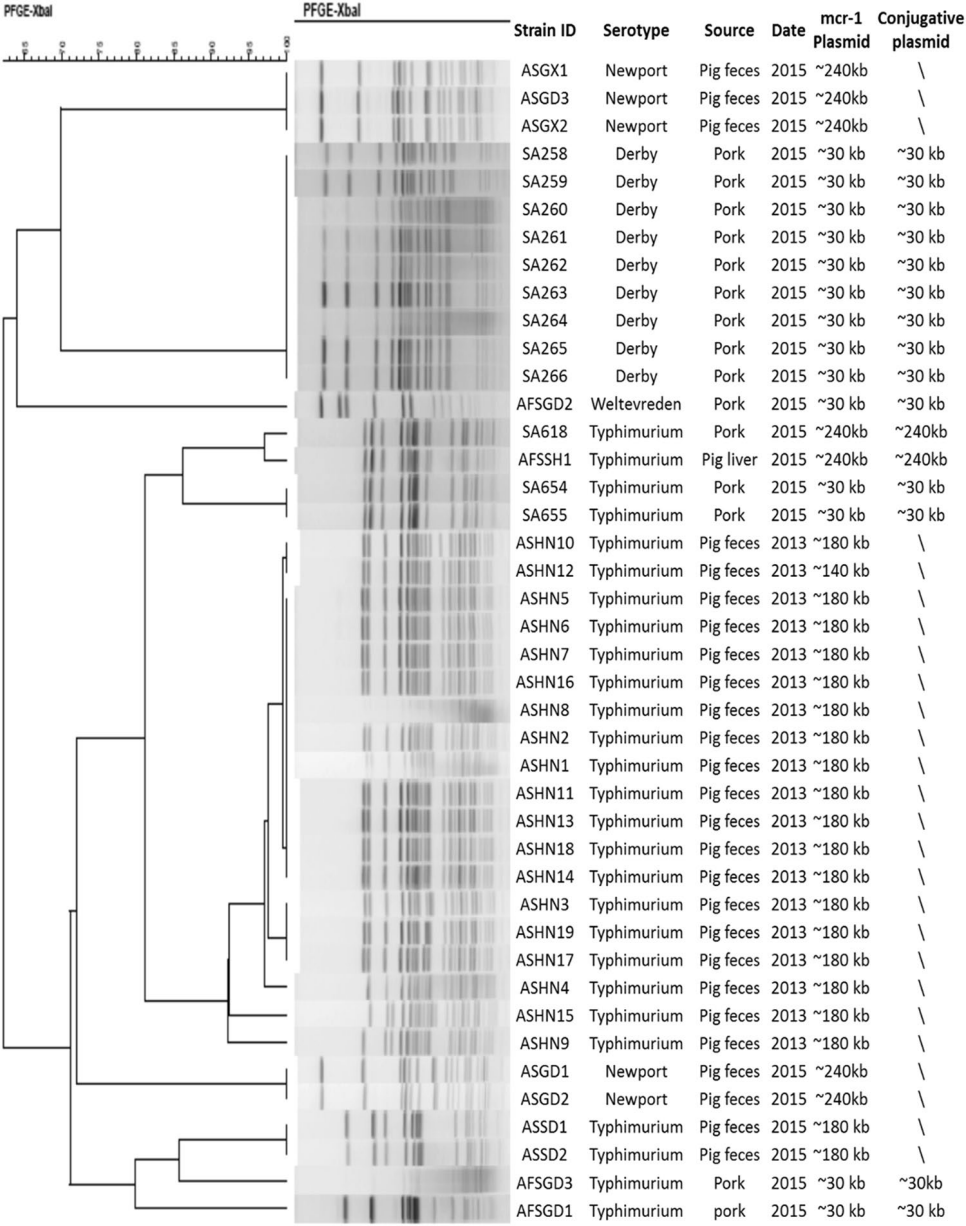
Summary of genetic characteristics of *mcr-1*-bearing *Salmonella* strains isolated from different sources. The isolation location and sources of the *Salmonella* isolates were designed as follow: ASHN1-ASHN19, *Salmonella* strains isolated from animal fecal samples from Henan province; ASSD1-ASSD2; *Salmonella* strains isolated from animal fecal samples from Shandong province; ASGD1-ASGD3, *Salmonella* strains isolated from animal fecal samples from Guangdong province; ASGX1-ASGX2, *Salmonella* strains isolated from animal fecal samples from Guangxi province; AFSGD1-AFSGD3, *Salmonella* strains isolated from food samples from Guangdong province; AFSSH1, *Salmonella* strains isolated from food samples from Shanghai province; SA258-SA266, *Salmonella* strains isolated from food samples in Shenzhen.

Most of the *mcr-1*-positive *Salmonella* strains were found to be *S*. Typhimurium. The 26 animal isolates included 21*S*. Typhimurium, and 5*S*. Newport. Among the 21*S*. Typhimurium strains, 19 were isolated in different locations in Henan province in 2013 and were found to exhibit almost identical PFGE pattern, suggesting that clonal dissemination of this specific clone in animals in Henan province had occurred; 2 were isolated from Shandong province in 2015 and exhibited identical PFGE pattern. Another 5 animal isolates belonged to *S*. Newport that were isolated from Guangdong and Guangxi provinces with two different PFGE patterns. Among the food-borne *Salmonella* isolates, 9 were S. Derby that belonged to the same clone and were isolated from different locations in Shenzhen on the same day in 2015; five were *S*. Typhimurium, among which three were isolated from Shenzhen with very similar PFGE pattern and two were from other parts of China with different PFGE patterns, and one was *S*. Welteweden. Interestingly, *mcr-1*-positive *Salmonella* isolated from animal and food showed very different serotypes and PFGE pattern. None of the food *Salmonella* isolate showed identical PFGE pattern as animal *Salmonella* isolate, which might suggest that the transmission of *mcr-1*-positve animal *Salmonella* to food products is not a common event (Fig. 1).

### Mechanisms of transmission of *mcr-1* among *Salmonella* of different sources

**Figure 2:**
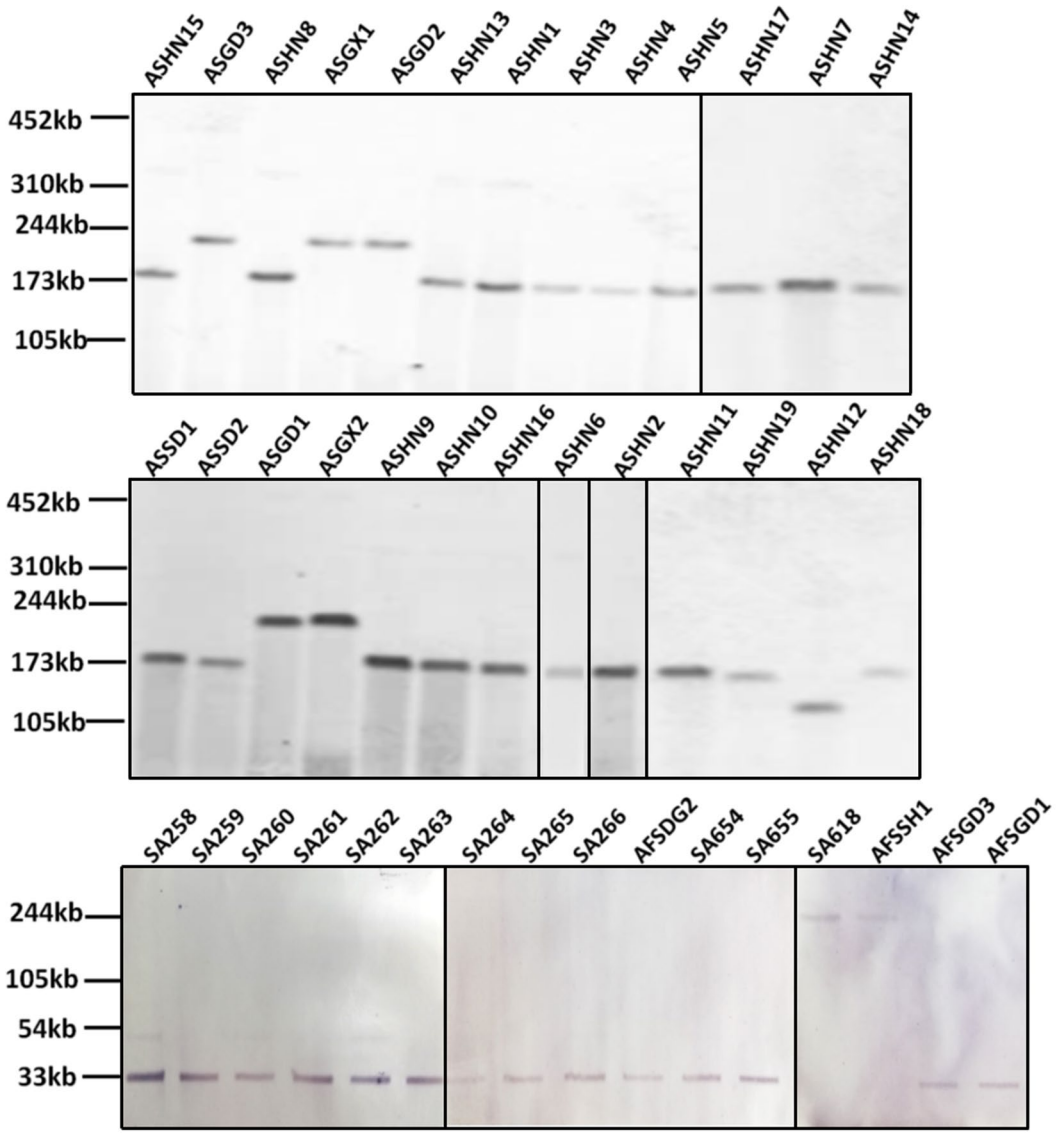
S1-PFGE and Southern Hybridization of all *Salmonella* isolates from animal and food. The results of hybridization to *mcr-1* probe was shown.

To investigate the transmission potential of *mcr-1* among *Salmonella* strains isolated from animal and food, all *mcr-1*-positive strains were tested for their ability to undergo conjugation. Surprisingly, strains isolated from different sources exhibited highly different conjugation rate: the *mcr-1* gene in all the animal isolates tested were not conjugative, while the gene in all food isolates were conjugative. S1-PFGE and Southern hybridization were then performed all animal *Salmonella* isolates and both parental strains and transconjugants of food isolates to investigate the genetic features of plasmids recoverable from these *Salmonella* strains. Among the 26 animal isolates tested, 20 were found to contain one ~180 kb *mcr-1*-bearing plasmid, 5 contained a ~240 kb plasmid and one contained a ~140 kb plasmid. Among the 16 *Salmonella* food isolates tested, conjugative plasmids of 30 kb and 240 kb in size were recoverable from 14 and 2 strains respectively (Figs 1, 2).

### Genetic features of *mcr-1*-encoding plasmids recovered from *Salmonella* strains isolated from different sources

To further delineate the genetic features of different conjugative and non-conjugative plasmids harboring the *mcr-1* element, representative plasmids were selected from different strains including two ~30 kb and one ~250 kb plasmid from food isolates, one each of ~140 kb and ~240 kb and two ~180 kb plasmids from animal isolates, for sequencing using the Illumina platforms. One ~180 kb plasmid recovered from strain ASSD2 was further sequenced by the PacBio platform to obtain the complete map (Table 2). Illumina contiges of two ~30 kb plasmids harbored by *Salmonella* strains, *S*. Weltevreden AFSGD2 and *S*. Derby SA258, were obtained and aligned to several previously reported plasmids, pOW3E1(KX129783.1) and pECJP-B65-33 (KX084392.1). These two plasmids were shown to belong to IncX4 type and could be aligned very well to pOW3E1(KX129783.1) ( > 99% in both identity and coverage) (Data not shown).

**Table 2:** Origin and genetic features of *mcr-1*-bearing plasmids in *Salmonella* strains subjected to sequence analysis in this study.

Strain ID	Serotype	source	Year of Isolation	S1-PFGE	CN	Plasmid types	Complete sequences/ contigs
AFSGD2	Weltervredn	PK	2014	~30 kb	C	IncX4	contigs
SA258	Derby	PK	2015	~30 kb	C	IncX4	contigs
ASGD2	Newport	PS	2015	~240 kb	NC	IncHI2	contigs
ASSD2	Typhimurium	PS	2015	~180 kb	NC	IncHI2	pASSD2-MCR1
ASHN8	Typhimurium	PS	2013	~180 kb	NC	IncHI2	contigs
ASHN12	Typhimurium	PS	2013	~140 kb	NC	IncHI2	contigs
SA618	Typhimurium	PS	2015	~250 kb	C	IncHI2	contigs

HS, human stool; PK, pork; PS, pig feces; CN, conjugative nature; C, conjugative; NC, non-conjugative.

**Figure 3:**
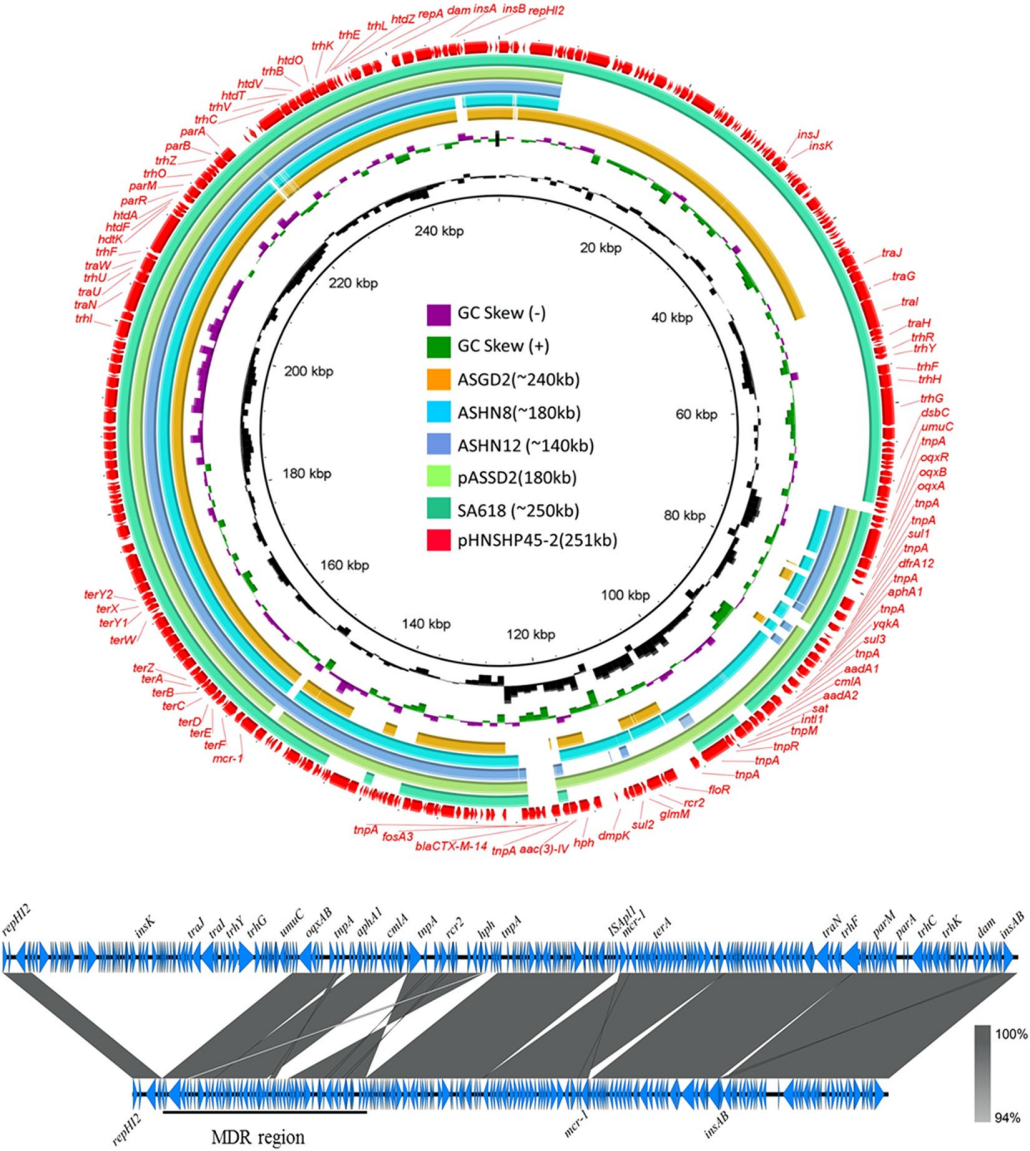
Alignment of conjugative and non-conjugative IncHI2 plasmids/contigs against pHNSHP45-2. (a) the circular map was created by BRIG tools; the linear map was generated by EasyFig. Genes in the reference plasmid, pHNSHP45-2, which was reported previously, are labeled by red arrows. Two other plasmids, pHSHLJ1-MCR1, pASSD2-MCR1 and contigs of other plasmids labeled with different colors were aligned to the reference plasmid. The gaps in the plasmid sequences represent the missing sequences when compared to the reference plasmid; (b) alignment of pASSD2-MCR1 to pHNSHP45-2 using Easyfig. The major different between these two plasmids is the absence of a gene fragment encoding tra genes that are responsible for plasmid conjugation was detected in pASSD2-MCR1 accounting for its smaller size compared to pHNSHP45-2. In addition, some variations were also detected in the MCR regions of these two plasmids. The other backbone regions in these two plasmids were almost identical.

**Figure 4:**
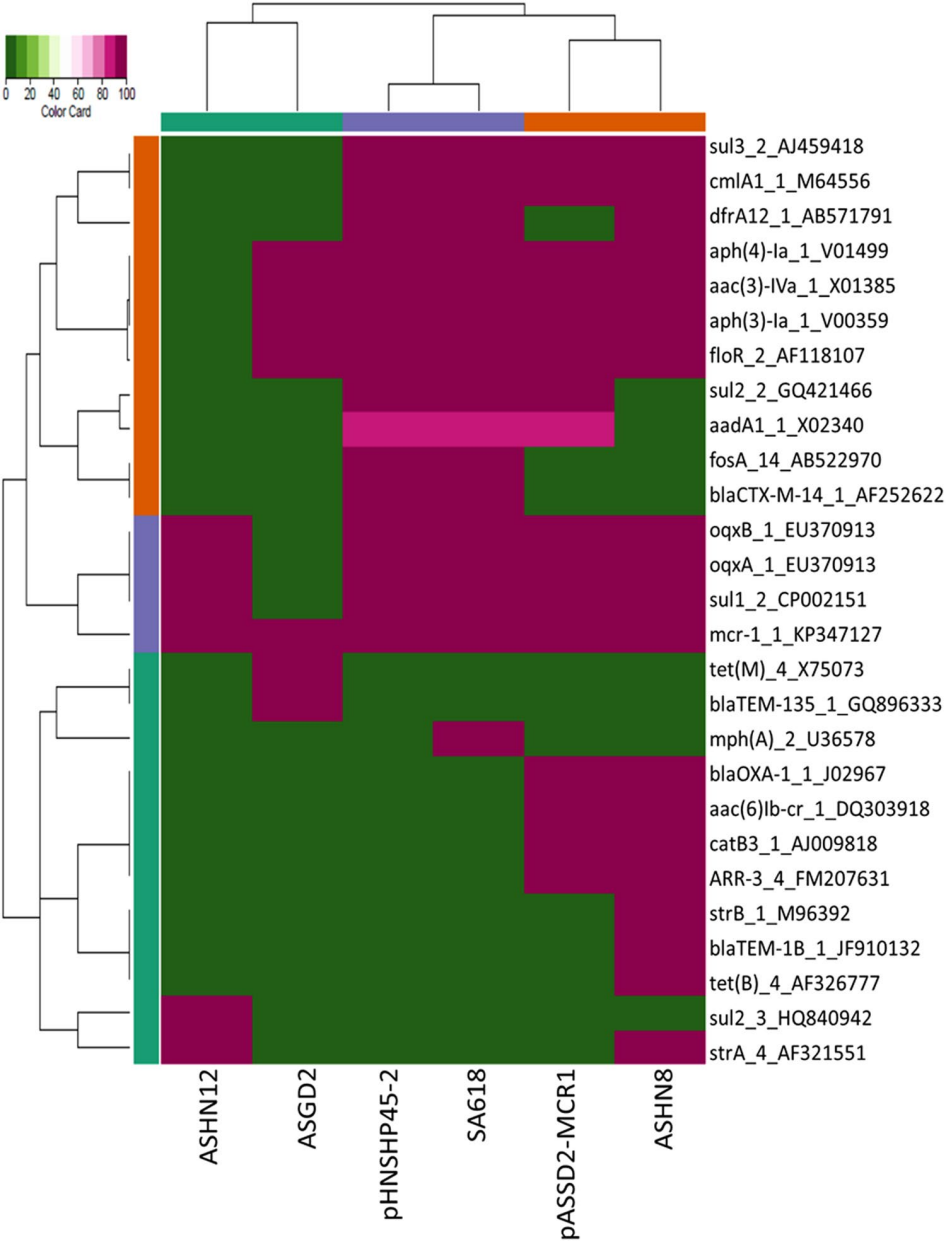
Antimicrobial resistance-related gene analysis for all IncHI2 plasmids with various sizes. Plasmid information was shown in Table 2.

The Illumina sequencing data showed that both conjugative or non-conjugative plasmids with sizes of ~140 kb, ~180 kb, ~240 kb and ~250 kb belonged to the IncHI2 type, which contained all or part of the previously reported ~251 kb plasmid pHNSHP45-2 (KU341381.1) (Fig. 3). The illumina sequence of a conjugative ~250 kb plasmid recovered from *S*. Typhimurium SA618 was obtained and compared to pHNSHP45-2, with results showing that this conjugative plasmid displayed a high degree of sequence homology to pHNSHP45-2 (Fig. 3). Antimicrobial resistance gene analysis showed that conjugative plasmid from SA618 contained one more *mphA(2)* gene (Fig. 4). Interestingly, alignment of Illumina reads of the non-conjugative ~240 kb plasmids recovered from the *S*. Newport strains ASGD2 to pHNSHP45-2 revealed that this plasmid aligned very well with pHNSHP45-2 except that a region containing the *tra* genes, which were responsible for conjugation, was absent in the plasmid of strain ASGD2, as well as some mobile elements in the Multidrug Resistance (MDR) Region was absent (Fig. 3). Antimicrobial resistance gene analysis showed that the plasmid from *S*. Newport strains ASGD2 lacked the mobile elements carrying *bla*_CTX-M-14_, *oqxAB*, *fosA3, aadA1* and other antimicrobial resistance genes such as *dfrA12* and *cmlA1*, but gained mobile elements carrying other antimicrobial resistance genes and transposase genes such as *tetM* and *bla*_TEM135_. Interestingly, different from pHNSHP45-2 that carried an intact Tn*6330*, the mobile element carrying *mcr-1* on this plasmid lacked the downstream IS*Apl1*. The lack of a *tra* region may explain why the ~240 kb plasmids of *Salmonella* animal isolates were non-conjugative (Figs 3, 4).

The complete sequence of one ~180 kb, non-conjugative plasmid recovered from the *S*. Typhimurium strain ASSD2, namely pASSD2-MCR1, was obtained and shown to be 187, 257 bp in size. This plasmid was found to belong to IncHI2 and exhibit high level homology to pHNSHP45-2. Detailed alignment between pHNSHP45-2 and the non-conjugative plasmid pASSD2-MCR1 revealed several different genetic rearrangements including: (i) the *mcr-1* gene without IS*Apl1* in the upstream in pASSD2-MCR1 was located in different sites compared to plasmid pHNSHP45-2; (ii) the mobile element *insAB* was repeatedly inserted in different locations in pASSD2-MCR1; (iii) the MDR region differed by one inversion; (iv) a large region of ca. 70 kb harboring the *tra* regions was absent in pASSD2-MCR1; (v) loss of mobile element carrying *bla*_CTX-M-14_ and *fosA3* and gaining a mobile element carrying *bla*_OXA-1_, *aac(6)-Ib-cr* and *catB3* (Figs 3, 4). These different genetic features, detectable among the two plasmids, depicted the evolution routes of the IncI2 class of *mcr-1*-carrying plasmids, and indicated that the loss of the ca. 70 kb *tra* region in pASSD2-MCR1 could contribute to the loss of transferability of this plasmid. The illumina sequence data of the other non-conjugative plasmids with sizes of ~180 kb recovered from *Salmonella* ASHN8 was shown to be highly homologous to pASSD2-MCR1(187 kb) and belong to the IncHI2 type plasmid, and that it obtained some more mobile elements carrying different antimicrobial resistance genes such as *StrAB*, *bla*_TEM_ and *tetB* (Fig. 4). Similar as the ~240 kb plasmid in this study, the mobile element carrying *mcr-1* on the ~180 kb plasmids also lacked the downstream *ISApl1*. The ~140 kb plasmid recovered from *S*. Typhimurium strain ASHN12 was very similar to pASSD2-MCR1(187 kb) in the plasmid backbone region, but with further deletions in the MDR region. It lacked almost all the mobile elements carrying antimicrobial resistance gene presence in pHNSHP45-2, but only remained the mobile element carrying *oqxAB* gene. It also acquired one more antimicrobial resistance genes, *StrA* gene compared to pHNSHP45-2 (Figs 3, 4). Interesting, plasmid from ASHN12 was found to carry the intact Tn*6330* as reported in plasmid, pHNSHP45-2, with two copies of IS*Apl1* located up- and down-stream of the *mcr-1* gene.

Genetic features of *mcr-1*-positive plasmids obtained from various sources were consistent with the antimicrobial resistance phenotypes of *Salmonella* isolates. All animal *Salmonella* isolates carried *mcr-1* plasmids without the *bla*_CTX-M-14_ and *fosA3* genes, therefore none of them was resistant to cephalosporins and fosfomycin. Two *Salmonella* isolates from food carried conjugative ~250 kb plasmids harboring *bla*_CTX-M-14_ and *fosA3* mobile elements, therefore these strains were resistant to cephalosporins and fosfomycin.

## Discussion

Compared to other bacterial species of *Enterobacteriaceae*, the prevalence rate of *mcr-1* in *Salmonella* was found to be much lower in each of these surveys, regardless of the site of recovery^16–20^. The low prevalence of *mcr-1* was also reported in a study from China, England and Wales^3,21^. However, another study in China reported the high prevalence of *Salmonella* from animals^9^. In addition, our data showed that most of the *Salmonella* strains that harbored *mcr-1*-bearing plasmids were *S*. Typhimurium, suggesting that *mcr-1*-bearing plasmids might have strong association with specific serotypes of *Salmonella*. The close association between *S*. Typhimurium and *mcr-1*-bearing plasmids entails further investigation. In animal and food *Salmonella* isolates, clonal spread of *mcr-1*-positive *Salmonella* was common. *S*. Derby strains isolated from food samples in Shenzhen, China were genetically identical, and were therefore likely the result of clonal dissemination of a single *mcr-1*-bearing strain. All the animal isolates could be grouped into three clusters: 19*S*. Typhimurium isolated in 2013 in Henan province exhibited similar PFGE patterns, five *S*. Newport strains isolated from 2015 belonged to two types of PFGE patterns and two *S*. Typhimurium isolated from 2015 were genetically identical. These data suggested that the *mcr-1*-positive *Salmonella* is similar from same location, while diverse between different part of China. However, it is interesting that *mcr-1*-positive *Salmonella* from food and animal did not share similar PFGE pattern, suggesting the less common event of transmission of *mcr-1*-positive *Salmonella* from animals to food samples.

This phenomenon was further supported by data on genetic analysis of *mcr-1*-bearing plasmids carried by *Salmonella* strains isolated from animals and food specimens. All *mcr-1*-bearing plasmids recovered from animal *Salmonella* isolates were non-conjugative with sizes of ~140 kb, ~180 kb or ~240 kb, the representative of which were shown to be IncHI2. However, all *mcr-1*-bearing plasmids from food *Salmonella* isolates were conjugative and belonged to two types, ~33 kb IncX4 and ~250 kb IncHI2, which exhibited identical sequences to plasmids of similar types recoverable from other bacterial species in the family of *Enterobacteriaceae*. These two types of plasmids were not detected in *Salmonella* isolates of animal origin, suggesting that they may be transmitted to *Salmonella* from other *mcr-1*-positive bacteria such as *E. coli* which co-exist in food but not in animal GI tract.

In conclusion, results of this nationwide surveillance of *mcr-1* and its transmission mechanisms in *Salmonella* provide comprehensive understanding of the features and mechanisms of transmission of *mcr-1* in *Salmonella* recovered from different settings, and hence lay the foundation for future development of strategies to control the transmission of this colistin resistance determinant among Gram negative bacterial pathogens.

## Materials and Methods

### Salmonella strains

*Salmonella* strains were collected from animals and food nationwide in China. All test strains were isolated in CHROMagar *Salmonella* agar (CHROMagar Company, Paris, France) and XLT4 agar (Oxoid). Suspected *Salmonella* colonies were selected for biochemical confirmation using the API 20 E system (bioMérieux, Marcy l’ Etoile, France), as well as via molecular identification by PCR assay targeting the *invA* gene, followed by sequencing. *Salmonella* serotyping was conducted by performing the slide agglutination test, using *Salmonella* antisera (S & A Reagents Lab Ltd., Bangkok, Thailand) according to the Kaufman-White scheme. All animal fecal samples were collected in accordance with relevant guidelines and regulations of the China Institute of Veterinary Drug Control, Beijing, P. R. China. All experimental protocols were approved by the China Institute of Veterinary Drug Control, Beijing, P. R. China.

### Screening of the *mcr-1* gene in *Salmonella*

*Salmonella* genomic DNA was prepared using the boiling method. PCR was performed using the primers targeting *mcr-1* as reported previously^16^. The genetic identity of all amplification products was confirmed by nucleotide sequencing.

### Antimicrobial susceptibility tests

All *Salmonella* isolates were subjected to antimicrobial susceptibility tests by the standard agar dilution method as described by the Clinical and Laboratory Standards Institute^22,23^. Fourteen antimicrobials as shown in Table 1 were tested. *Escherichia coli* strain ATCC 25922 was used as the quality control.

### Conjugation, PFGE, S1-PFGE and Southern Hybridization

Conjugation experiments were carried out using the mixed broth method as previously described^24^. PFGE, S1-PFGE and Southern Hybridization were performed as previously described^25^.

### Plasmid sequencing

Representative plasmids with sizes of ~33 kb, ~60 kb, 140 kb ~180 kb and ~240 kb, recovered from *Salmonella* parental strains and transconjugants, were subjected to plasmid sequencing using the Illumina and PacBio platforms and analysed as previously described^26^. The complete nucleotide sequences of the ~180 kb plasmid, pASSD2-MCR1(KX856065), were submitted to GenBank.
